# Locus-specific view of flax domestication history

**DOI:** 10.1002/ece3.57

**Published:** 2012-01

**Authors:** Yong-Bi Fu, Axel Diederichsen, Robin G Allaby

**Affiliations:** 1 Plant Gene Resources of Canada, Saskatoon Research Centre, Agriculture and Agri-Food Canada107 Science Place, Saskatoon, SK S7N 0X2, Canada; 2 School of Life Sciences, University of WarwickWellesbourne, Warwick CV35 9EF, United Kingdom

**Keywords:** Crop domestication, flax, network analysis, *sad2* gene, sequence variation

## Abstract

Crop domestication has been inferred genetically from neutral markers and increasingly from specific domestication-associated loci. However, some crops are utilized for multiple purposes that may or may not be reflected in a single domestication-associated locus. One such example is cultivated flax (*Linum usitatissimum* L.), the earliest oil and fiber crop, for which domestication history remains poorly understood. Oil composition of cultivated flax and pale flax (*L. bienne* Mill.) indicates that the *sad2* locus is a candidate domestication locus associated with increased unsaturated fatty acid production in cultivated flax. A phylogenetic analysis of the *sad2* locus in 43 pale and 70 cultivated flax accessions established a complex domestication history for flax that has not been observed previously. The analysis supports an early, independent domestication of a primitive flax lineage, in which the loss of seed dispersal through capsular indehiscence was not established, but increased oil content was likely occurred. A subsequent flax domestication process occurred that probably involved multiple domestications and includes lineages that contain oil, fiber, and winter varieties. In agreement with previous studies, oil rather than fiber varieties occupy basal phylogenetic positions. The data support multiple paths of flax domestication for oil-associated traits before selection of the other domestication-associated traits of seed dispersal loss and fiber production. The *sad*2 locus is less revealing about the origin of winter tolerance. In this case, a single domestication-associated locus is informative about the history of domesticated forms with the associated trait while partially informative on forms less associated with the trait.

## Introduction

Genetic studies of crop domestication have increased in the last two decades, largely thanks to the development of many informative molecular techniques (Zeder et al. 2006; Burke et al. 2007; Purugganan and Fuller 2009). Considerable research has been performed to investigate domestication events using selectively neutral and genome-wide molecular markers (Heun et al. 1997; Badr et al. 2000; Matsuoka et al. 2002; Morrell and Clegg 2007; Fu 2011). However, there has been an increasing trend to reconstruct the evolutionary history of domestication through the loci that have been subject to selection (Sang 2009; Gross and Olsen 2010; Blackman et al. 2011), as the genetic bases have been uncovered for many domestication-associated traits, including plant structural changes (Wang et al. 1999; Doebley et al. 2006; Li et al. 2006) and food quantity and quality (Sweeney et al. 2007; Shomura et al. 2008; Kovach et al. 2009). In the case of plants, the domestication process is increasingly considered to have been a protracted process (Allaby et al. 2008), and the assemblage of domestication-associated traits a staggered process (Fuller 2007). Under this scenario, there is an increased likelihood that each trait may have a quite disparate evolutionary history (Allaby 2010). Crops that have multiple purposes are interesting in this respect because genes governing traits relating to specific groups of the crop may carry variable domestication signatures. Thus, a locus-specific inference of different trait groups should reveal not only the group-specific domestication history but also the correlated domestication processes.

Flax (*Linum usitatissimum* L.) is a good example of a multiple purpose crop being utilized for oil and fiber. It was one of the eight “founder crops” of agriculture, was a principal source of oil and fiber from prehistoric times until the early 20th century, and still remains a crop of considerable economic importance (Zohary and Hopf 2000; Muir and Westcott 2003). The archaeological record shows that flax was domesticated for oil and/or fiber use more than 8000 years ago in the Near East (Helbaek 1959; van Zeist and Bakker–Heeres 1975) and suggests its wild progenitor as pale flax (*L. bienne* Mill. or previously *L. usitatissimum* L. subsp. *angustifolium* (Huds.) Thell.; Hammer 1986). The earliest reliable evidence of pale flax for human usage comes from Tell Abu Hureyra 11,200–10,500 years before present (yBP) (Hillman 1975), although recent claims have been made for much earlier usage that are disputed (Kvavadze et al. 2009; Bergfjord et al. 2010).

Morphological, cytological, and molecular characterizations confirm that pale flax is the wild progenitor of cultivated flax (Tammes 1928; Gill 1966, 1987; Diederichsen and Hammer 1995; Fu et al. 2002; Fu and Allaby 2010). Pale flax is a winter annual or perennial plant with narrow leaves and dehiscent capsules, and usually displays large variation in the vegetative plant parts and variable growth habit (Diederichsen and Hammer 1995; Uysal et al. 2011). Recent studies that expanded the available pale flax germplasm (Uysal et al. 2010, 2011) are informative about flax domestication syndromes (Hammer 1984). Generally, cultivated flax has variable seed dormancy, grows fast with large variation in the generative plant parts, and has early flowering, almost indehiscent capsules, and large seeds. In addition to oil and fiber varieties, flax accessions with winter hardiness and capsular dehiscence are also available for research (Diederichsen and Fu 2006).

Domestication-associated genes offer an approach to reconstructing the specific domestication history of a crop with an associated trait with phylogenetic and phylogeographic resolution. Such approaches have provided insights into the independent origins of various traits in rice (e.g., Shomura et al. 2008; Kovach et al. 2009) and in sunflower (Blackman et al. 2011). However, flax presents several problems for this type of approach. First, no domestication-associated genes have yet been identified in flax. Second, the variety of uses of flax implies that different subsets of cultivated flax have different trait combinations, and consequently it is not clear to what extent a single domestication gene may be used to infer the domestication history of the crop as a whole.

The *sad2* locus is a potential domestication target in flax because of its role in fatty acid metabolism. The *sad2* gene is responsible for converting stearoyl-ACP to oleoyl-ACP by introducing a double bond at C_9_ and thus can increase the unsaturated fatty acid content of the plant (Ohlrogge and Jaworski 1997; Jain et al. 1999). This gene has been well characterized due to commercial interest for the manipulation of unsaturated fatty acids in major crop plants (Shanklin and Sommerville 1991; Knutzon et al. 1992; Singh et al. 1994). Preliminary molecular evidence based on the *sad2* locus in a relatively small sample of flax suggested that the initial purpose of flax domestication was for its oil use (Allaby et al. 2005). This study was limited because it considered very few pale flax accessions and only oil, fiber, and landrace varieties of cultivated flax. More recently, expressed sequence tag-derived simple sequence repeat (EST-SSR) markers (Cloutier et al. 2009; Fu and Peterson 2010) were applied to the expanded pale flax germplasm (Fu 2011). This study established that the primitive dehiscent type of cultivated flax assumed a basal position in genome-wide marker phenograms, suggesting that these varieties were important in the early stages of domestication. However, the overall resolution was not high.

The aim of this study was to assess whether the *sad2* locus increases oil production in cultivated flax and whether a reconstruction of the domestication history of this trait based on the gene is widely informative for cultivated flax using a broad sample of accessions representing the recently expanded pale flax germplasm set and the four cultivated flax groups.

## Materials and Methods

All flax accessions studied here were obtained from the flax collection at the Plant Genetic Resources of Canada (PGRC; Table 1). They include 43 pale flax accessions and 70 cultivated flax accessions. The pale flax accessions were selected largely from recently acquired pale flax accessions from Turkey and Greece, in addition to those representing the old pale flax collection in PGRC. This expanded set of pale flax accessions represent only part of its natural distribution spanning the western Europe and the Mediterranean, north Africa, western and southern Asia, and the Caucasus regions (Diederichsen and Hammer 1995). The cultivated flax accessions were selected to represent five major groups of cultivated flax (landrace, fiber, oil, winter, and dehiscent). The landrace group represents a collection of local oil and/or fiber varieties from different countries. The winter flax accessions sampled cultivated flax developed with winter hardiness from 12 countries. The dehiscent flax accessions represent the primitive form of cultivated flax with dehiscent capsules and have been long accumulated from flax cultivation in the cultivated flax gene pool (Hegi 1925). For this study, the dehiscent flax accessions were empirically verified for capsular dehiscence and the selected pale flax accessions were assessed for their taxonomic identity in the greenhouse. Also, the accession selection process took into account the country of origin to widen genetic diversity for this study.

**Table 1:** List of 113 accessions of wild and cultivated flax sequenced, with their species/type and origin country

CN^1^	Species/type^2^	Description^3^	Origin^4^	Label^5^	CN	Species/type	Description	Origin	Label
107293	Lb		UN(1)	Bm1	98475	Lu-f	Flachskopf	DEU	Uf7
107257	Lb		UN(2)	Bm2	101392	Lu-f	Tajga	FRA	Uf8
19021	Lb		FRA	Bm3	101111	Lu-f	Viking	FRA	Uf9
107258	Lb		UN(2)	Bm4	101086	Lu-f	Ariadna	HUN	Uf10
19022	Lb		DEU	Bm5	98946	Lu-f	Talmune fiber	NLD	Uf11
19023	Lb		USA	Bm6	101120	Lu-f	Liana	POL	Uf12
113601	Lb	Samsun	TUR	Bt1	97325	Lu-f	Kotowiecki	POL	Uf13
113602	Lb	Samsun	TUR	Bt2	101405	Lu-f	Mures	ROM	Uf14
113603	Lb	Samsun	TUR	Bt3	18991	Lu-f	Nike	RUS	Uf15
113604	Lb	Samsun	TUR	Bt4	97871	Lu-f	Atlas	SWE	Uf16
113605	Lb	Samsun	TUR	Bt5	101397	Lu-f	Pskovski 2976	UKR	Uf17
113606	Lb	Samsun	TUR	Bt6	101021	Lu-n	Mestnyi	AFG	Un1
113607	Lb	Samsun	TUR	Bt7	19009	Lu-n	Mestnyi	CHN	Un2
113608	Lb	Samsun	TUR	Bt8	100896	Lu-n	Giza	EGY	Un3
113610	Lb	Denizli	TUR	Bt9	100895	Lu-n	Karbin	ETH	Un4
113616	Lb	İzmir	TUR	Bt10	100890	Lu-n	Svapo	FRA	Un5
113617	Lb	İzmir	TUR	Bt11	19010	Lu-n	Mestnyi	IRN	Un6
113618	Lb	Muğla	TUR	Bt12	100909	Lu-n	Palestina	ISR	Un7
113619	Lb	Muğla	TUR	Bt13	100911	Lu-n	Cremone	ITA	Un8
113620	Lb	Muğla	TUR	Bt14	101070	Lu-n	landrace	RUS	Un9
113621	Lb	Muğla	TUR	Bt15	101614	Lu-o	Signal	BLR	Uo1
113622	Lb	Antalya	TUR	Bt16	19003	Lu-o	AC McDuff	CAN	Uo2
113623	Lb	Antalya	TUR	Bt17	18974	Lu-o	CDC Bethune	CAN	Uo3
113626	Lb	Samsun	TUR	Bt18	100832	Lu-o	Barbarigo	CZE	Uo4
113627	Lb	Sinop	TUR	Bt19	101174	Lu-o	Rastatter	DEU	Uo5
113628	Lb	Karabük	TUR	Bt20	97436	Lu-o	Giza	EGY	Uo6
113629	Lb	Kastamonu	TUR	Bt21	101171	Lu-o	Hermes	FRA	Uo7
113630	Lb	Kastamonu	TUR	Bt22	18989	Lu-o	Atalante	FRA	Uo8
113632	Lb	Zonguldak	TUR	Bt23	101265	Lu-o	Amason	GBR	Uo9
113633	Lb	Zonguldak	TUR	Bt24	98263	Lu-o	Chaurra Olajlen	HUN	Uo10
113634	Lb	Bolu	TUR	Bt25	98256	Lu-o	Arreveti	IND	Uo11
113635	Lb	Bolu	TUR	Bt26	97888	Lu-o	Tomagoan	IRN	Uo12
113636	Lb	Bilecik	TUR	Bt27	101237	Lu-o	Artemida	LTU	Uo13
113637	Lb	Bursa	TUR	Bt28	101268	Lu-o	Raisa	NLD	Uo14
113638	Lb	Çanakkale	TUR	Bt29	101245	Lu-o	Bryta	POL	Uo15
113639	Lb	Çanakkale	TUR	Bt30	100917	Lu-o	Raluga	ROM	Uo16
113640	Lb	Istanbul	TUR	Bt31	101233	Lu-o	Rolin	ROM	Uo17
113641	Lb	Çanakkale	TUR	Bt32	101292	Lu-o	Zarjanka	RUS	Uo18
113642	Lb	Trabzon	TUR	Bt33	98965	Lu-o	New River	USA	Uo19
T19719	Lb	Island of Evia	GRC	Bg1	33399	Lu-o	Bison	USA	Uo20
T19718	Lb	Island of Evia	GRC	Bg2	98178	Lu-w	1285-S	AFG	Uw1
T19717	Lb	Island of Koss	GRC	Bg3	97756	Lu-w	Italia Roma	ARG	Uw2
T19716	Lb	Rhodes airport	GRC	Bg4	96915	Lu-w	Uruguay 36/49	AUS	Uw3
97606	Lu-d		ESP	Ud1	97015	Lu-w	Uruguay 36/49	AUS	Uw4
100852	Lu-d	Grandal	PRT	Ud2	97009	Lu-w	Beladi Y 6903	EGY	Uw5
100910	Lu-d	Grandal	PRT	Ud3	97004	Lu-w		ETH	Uw6
97769	Lu-d	Abertico	PRT	Ud4	97205	Lu-w	Redwing 92	GRC	Uw7
97473	Lu-d		RUS	Ud5	98283	Lu-w	La Previzion	HUN	Uw8
98833	Lu-d		RUS	Ud6	98509	Lu-w		ISR	Uw9
97605	Lu-d		RUS	Ud7	97102	Lu-w		PAK	Uw10
100837	Lu-d		TUR	Ud8	96846	Lu-w		RUS	Uw11
101160	Lu-f	Wiko	AZE	Uf1	96960	Lu-w		SYR	Uw12
98986	Lu-f	Crista	BEL	Uf2	96848	Lu-w		TUR	Uw13
98935	Lu-f	Motley fiber	BLR	Uf3	96902	Lu-w		TUR	Uw14
101017	Lu-f	Baladi	CHN	Uf4	100828	Lu-w		TUR	Uw15
98479	Lu-f	Zakar	CZE	Uf5	100829	Lu-w		TUR	Uw16
101388	Lu-f	Saskai	CZE	Uf6					

1 CN = Canadian National accession number at Plant Gene Resources of Canada (PGRC), Saskatoon, Canada. T = temporary number for accessions that were acquired, but not yet added to the PGRC germplasm collection.

2 Lb = *Linum bienne*; Lu = *Linum usitatissimum*. Five letters (n, f, o, w, d) after Lu represents five groups of cultivated flax (landrace, fiber, oil, winter, dehiscence), respectively.

3 Description of an accession includes the record, if available, for varietal or local name, location, and feature.

4 Origin of country, following ISO 3166–1 alpha-3 country code. UN = unknown origin, but the seed source is shown with a number in parentheses: 1 = All-Russian Flax Research Institute, VNIIL, Torzhok, Russia, and 2 = Jardin Botanique de la Ville et de l’Universite de Caen, France.

5 Accession label includes the first letter for species (B = *L. bienne*; U = *L. usitatissimum*), the second letter (if any) for the country of *L. bienne* accessions (t = Turkey, g = Greece, and m = multiple countries) and for the group of cultivated flax (n = landrace, f = fiber, o = oil, w = winter, d = dehiscence), and the number distinguishing among accessions.

### Oil profile

The oil profile data used in this study were collected from two separate characterization efforts. The first one was completed before 2006 on 2934 accessions of cultivated flax (Diederichsen and Raney 2006) and the second one was performed in 2010 on 141 samples of pale flax by Drs. A. Diederichsen and R. Zhou. The pale flax samples largely consisted of the pale flax germplasm recently collected from Turkey and Greece. Both characterizations employed the same experimental procedures as described in Diederichsen and Raney (2006). Briefly, the seed oil content was measured using continuous wave nuclear magnetic resonance spectroscopy based on a sample of 10 g of flax seed at 3–4% water content. The fatty acid composition of the seed oil was analyzed by gas chromatography.

### DNA extraction

Plants were grown from seed for 2–3 weeks for cultivated flax and up to 2 months for pale flax in a greenhouse at the Saskatoon Research Centre, Agriculture and Agri-Food Canada. Young leaves were individually collected, freeze-dried [in a Labconco Freeze Dry System (Kansas City, MO, USA) for 1–3 days], and stored at –20°C. A freeze-dried leaf sample of one individual plant from each accession was selected, and its genomic DNA was extracted with the DNEasy Plant Mini kit (Qiagen, Mississauga, Ontario, Canada). Extracted DNA was quantified with a Thermo Scientific NanoDrop 8000 spectrometer (Fisher Scientific Canada, Toronto, Ontario, Canada).

### PCR and sequencing

The protocols and procedures to amplify and to sequence the *sad2* locus were given in Allaby et al. (2005). Briefly, two sets of PCR primer pairs were applied to amplify the whole region of the *sad2* locus. PCR was performed on either a DYAD or PTC-200 thermocycler (Bio Rad, Mississauga, Ontario, Canada) and the PCR products were separated on 2% agarose (Sigma, Oakville, Ontario, Canada). Amplicons were excised from agarose gel, purified using a QiaQuick Gel purification kit (Qiagen), and resuspended in 16-µl Qiagen elution buffer. Sequencing was done using an Applied Biosystems capillary DNA sequencer (DNA Technologies Unit, Plant Biotechnology Institute, National Research Council of Canada, Saskatoon, Saskatchewan, Canada).

### Sequence analysis

All sequencing products were assembled with Vector NTI Suite's ContigExpress v9.0.0 (Invitrogen, Carlsbad, CA) and aligned using MUSCLE v3.6 (Edgar 2004). All aligned sequences were deposited into GenBank under accessions JN653341-JN653453. Population genetic analyses of aligned DNA sequences were performed using DnaSP program (Librado and Rozas 2009). Several measures of sequence variation were obtained, and they are the number of segregating sites, haplotype number, nucleotide diversity (π; Tajima 1983), the signal of selection (i.e., deviation from neutrality; Tajima 1989; Fu and Li 1993), and the frequency of recombination (i.e., the minimum number of recombination events; Hudson and Kaplan 1985). The comparative diversity analyses were also done for various groups of flax germplasm. Haplotype analyses with and without gaps and indels were performed using DnaSP program. The positions of SNPs and indels for each haplotype were generated.

An analysis of molecular variance (AMOVA) was also performed using Arlequin v3.01 (Excoffier et al. 2005) to quantify nucleotide variation between species and among various groups of *Linum* accessions. Three models of genetic structuring were considered: pale versus cultivated flax, two originating groups of pale flax, and five groups of cultivated flax. The significance of variance components and intergroup genetic distances for each model was tested with 10,010 random permutations.

A network analysis was applied to display phylogenetic relationships among taxa because this approach allows for extant ancestral states in which taxa occupy internal node positions, and reticulate relationships caused by character conflict, such as those resulting from recombination events. Briefly, networks provide a graphical approach to describing character conflict, instances where characters support different trees, as reticulations. The resulting graphs may then be interpreted as either containing all the most parsimonious trees, or as a visualization of recombination events. The phylogenetic network of the studied accessions was constructed as described previously (Allaby and Brown 2001; Allaby et al. 2005). A deletion of 46 nucleotides occurred at position 562 of the alignment, which was used as a character in building the network. The phylogenetic topology of the network was confirmed through maximum likelihood (fastDNAml; Olsen et al. 1994), neighbor-joining analyses (NEIGHBOR; Felsenstein 1989), and NeighborNet (SplitsTree; Huson and Bryant 2006).

The date estimates for nodes from the network were corroborated using the Bayesian MCMC approach implemented in BEAST v1.4 (Drummond and Rambaut 2007). Maximum clade credibility (MCC) phylogenies were generated using the node II/III split calibrated under a uniform prior with a range of 11,000–9500 yBP, under a GTR model with gamma distribution for site heterogeneity and a relaxed uncorrelated lognormal clock. Three tree prior models were investigated: (1) with tree prior as constant size; (2) with tree prior as expansion growth; and (3) with tree prior as exponential growth. The rest of the options were applied with default values. The Bayesian MCMC approach should be more informative for dating a lineage involving recombination events, as it directly calculates ultrametric phylogenies based only on sequence data and model parameters and incorporates both the branch length errors and the topological uncertainties (Rutschmann 2006).

## Results and Discussion

### Oil profile

**Figure 1:**
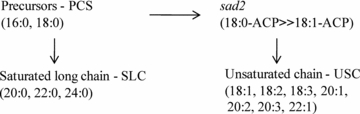
A simplified fatty acid pathway showing the sources and sinks for the function of *sad2* gene.

A considerably higher ratio of 18:1 oleoyl-ACP to precursor saturated fatty acids was obtained for the assayed groups of cultivated flax than the pale flax samples (Table 2). The ratio of total unsaturated to saturated precursor fatty acids, although not statistically significant, was generally higher in the cultivated, than pale, flax samples. These two sets of oil data can be interpreted as either an increase in total unsaturated fatty acids, or a decrease in the saturated acid precursors, or both. Figure 1 illustrates the salient features of fatty acid metabolism considered here. While an increase in the unsaturated fatty acid products would naturally be expected to lead to a decrease in unsaturated precursors, a second sink for the latter occurs through the production of long-chain saturated acids. However, the ratio of long-chain saturated fatty acids to the precursors is generally less in the cultivated, than pale, flax samples (Table 2), indicating that this path did not explain the decrease in precursor saturated fatty acids and indeed less long chain saturated fatty acids were produced in cultivated flax. Thus, there was an increase in the product of the *sad2* locus, 18:1 oleoyl-ACP. Such increase could be either due to a higher productivity of the *sad2* locus or a decrease in productivity downstream in the metabolic pathway at loci such as *fad2*, *fad3*, or *fae1*, so causing an accumulation of oleoyl-ACP. However, if the latter were to entirely explain the high levels of oleoyl-ACP, one would not expect the increase in the overall unsaturated to saturated fatty acid ratio observed. The downstream products in the metabolic pathway after the production of oleoyl-ACP are present in quantities approximately 20-fold higher than oleoyl-ACP. Thus, it is not surprising that the shift in ratio to saturated precursors is less pronounced for unsaturated acids as a whole than for just oleoyl-ACP. The oil composition data support that an increased productivity of the *sad2* locus in cultivated flax was associated with the increased unsaturated fatty acid content. Based on this oil profile, we can reason that the *sad2* locus is a candidate domestication-associated locus.

**Table 2:** Ratios of related fatty acid components for *Linum* groups

Group	*n*	18:1/PCS^1^	USC/PCS^1^	SLC/PCS × 100^1^
Pale flax	141	0.349(0.057)	9.055(0.855)	4.917(0.729)
Cultivated flax	2768	0.447(0.052)	9.431(1.844)	4.075(0.437)
Dehiscent flax	6	0.453(0.025)	9.992(0.855)	4.762(0.526)
Fiber flax	331	0.455(0.057)	10.643(1.686)	4.044(0.400)
Oil flax	2264	0.442(0.049)	9.373(1.794)	4.064(0.440)

1 PCS = precursor saturated fatty acids (16:0, 18:0), USC = unsaturated fatty acids (18:1, 18:2, 18:3, 20:1, 20:2, 20:3, 22:1), SLC = saturated long-chain fatty acids (20:0, 22:0, 24:0). The values in parentheses are standard errors of the ratio estimates. See Figure 1 for the simplified fatty acid pathway for related components.

### Nucleotide polymorphism

**Figure 2:**
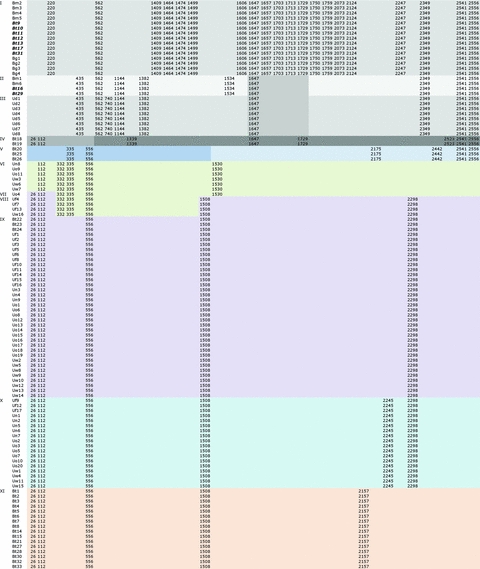
Composition of 39 polymorphic sites for each sample of 11 groups reflecting 11 haplotypes. The group is labeled in the first column, the sample in the second column (see Table 1), and the composition in the columns 3–41. The pale flax samples from western Turkey are highlighted with italic and bold. The numbers in the composition columns are the positions of substitutions. Different background colors were used to make the haplotype identification easier.

The aligned nucleotide sequences of *sad2* amplified from 113 accessions are 2560 bp in length, covering three exons, two introns, and the upstream and downstream flanking regions (Table 3). A total of 38 polymorphic sites were found with 17 segregating sites in intron 2 (45%), 12 sites in exon 3 (32%), six sites in intron 1 (16%), and one for each of other two exons and flanking region. All of them are parsimony informative. Only one indel of length 46 bp in the intron 1 at the positions from 562 to 607 was detected in all eight accessions of dehiscent flax and 19 pale flax accessions of diverse country origins including five accessions from Turkey. Interestingly, such an indel was not found in the other 24 pale flax accessions collected from Turkey and the remaining 62 accessions of cultivated flax. These findings support the existence of two distinctive backgrounds in pale flax germplasm collected from Turkey (Uysal et al. 2011). A large set of pale flax accessions was more closely related to cultivated flax (Fig. 2). An overall average pairwise nucleotide diversity of 0.00371 was obtained across all 113 samples. Deviation from neutrality was not significant with Tajima's *D* of 0.8856 (at *P* > 0.10), but significant by Fu and Li's D* and F* test statistics (D* = 2.0778 at *P* < 0.02 and F* = 1.9155 at *P* < 0.05, respectively; Fu and Li 1993). There were 13 synonymous mutations observed in all three exons, but only one nonsynonymous change at exon 3 (at position 2245) from serine to proline in the *sad2* protein was detected in cultivated flax. Such a nonsynonymous change was also observed in the Genbank accession AJ006958.

**Table 3:** Nucleotide polymorphism at the *sad2* locus for 10 groups of wild and cultivated flax samples

Group/parameter^1^	Flanking (187)^2^	Exon1 (123)	Intron1 (609)	Exon2 (505)	Intron2 (722)	Exon3 (563)	Total (2709)
Lb-all (43)							
S	1	1	4	1	16	11	34
H	2	2	5	2	5	6	6
π/bp	0.00701	0.00416	0.00228	0.00034	0.00918	0.00729	0.00515
*D*	1.6980	1.6980	0.9087	–0.3533	2.4867 3	1.5727	2.2276 3
Lb-Turkey (33)							
S	1	1	4	1	16	11	34
H	2	2	5	2	5	6	6
π/bp	0.00654	0.00388	0.00194	0.00023	0.0072	0.00676	0.00435
*D*	1.4168	1.4168	0.2676	–0.7915	1.0383	1.0290	1.0918
Lb-Others (10)							
S	0	0	2	1	13	3	19
H	1	1	2	2	2	2	2
π/bp	0	0	0.00126	0.0007	0.00644	0.00201	0.00269
*D*	nd	nd	0.0189	0.015	0.0266	0.0211	0.0274
Lu-all (70)							
S	1	1	5	1	4	5	17
H	2	2	3	2	3	4	6
π/bp	0.00649	0.00167	0.00205	0.00041	0.0013	0.00253	0.00167
*D*	0.6984	–0.0128	0.2534	–0.0128	0.2674	0.6723	0.5392
Lu-d (8)							
S	0	0	0	0	0	0	0
H	1	1	1	1	1	1	1
π/bp	0	0	0	0	0	0	0
*D*	nd	nd	nd	nd	nd	nd	nd
Lu-o (20)							
S	1	0	2	0	2	2	7
H	2	1	2	1	2	3	4
π/bp	0.0026	0	0.00088	0	0.00075	0.00134	0.00077
*D*	–0.5916	nd	–0.1119	nd	–0.1119	0.6105	0.0010
Lu-f (17)							
S	0	0	2	0	0	1	3
H	1	1	2	1	1	2	3
π/bp	0	0	0.00101	0	0	0.00058	0.00036
*D*	nd	nd	0.1100	nd	nd	0.0851	0.1248
Lu-w (16)							
S	1	0	2	0	2	2	7
H	2	1	2	1	2	3	4
π/bp	0.0065	0	0.00131	0	0.00091	0.00136	0.00099
*D*	0.1557	nd	0.8377	nd	0.2007	0.5192	0.6522
Lu-n (9)							
S	1	0	2	0	2	2	7
H	2	1	2	1	2	3	3
π/bp	0.00304	0	0.00073	0	0.00062	0.00146	0.00074
*D*	–1.0882	nd	–1.3624	nd	–1.3624	0.1959	–1.1893

1 Lb = *Linum bienne*; Lu = *Linum usitatissimum*; Lb-all for all wild flax samples; Lb-Turkey for wild flax samples from Turkey; Lb-others for wild flax samples from other countries; Lu-all for all cultivated flax samples; and five other groups of cultivated flax (landrace, fiber, oil, winter, dehiscent) labeled with five letters (n, f, o, w, d) after Lu, respectively. Four polymorphism parameters are S for the number of segregating sites; H for the haplotype number; π/bp for nucleotide diversity; and *D* for selection test by Tajima's *D*.

2 The length of the region(s) is given in parentheses. nd means not defined.

3 For the level of test significance at *P* < 0.05 for selection.

Further examination of nucleotide polymorphism between two species and among various groups of flax accessions revealed several more patterns of genetic diversity at the locus (Table 3). First, as expected, there was more genetic variation in pale flax than cultivated flax. The pale flax had 34 polymorphic sites with a nucleotide diversity of 0.00515, while the cultivated flax had 17 polymorphic sites with a nucleotide diversity of 0.00167. There were 14 polymorphic sites shared by two species, 20 unique to pale flax, and only four unique to cultivated flax (Fig. 2). Second, a significant deviation from neutrality was found only in intron 2 with Tajima's D of 2.4867, resulting in an overall significant selection observed in 43 pale flax accessions. However, such neutrality deviation disappeared when pale flax accessions were separated based on country origin between Turkey and other countries. Clearly, the pale flax accessions from Turkey had more variation than those from other countries with nucleotide diversity values of 0.00435 and 0.00274, respectively. Third, among various groups of cultivated flax, winter flax had the largest nucleotide diversity at the locus (0.00099) with four haplotypes, followed by the oil flax (0.00077) with four haplotypes, landrace flax (0.00074) with three haplotypes, and fiber flax (0.00036) with three haplotypes. All eight dehiscent flax samples had the same haplotype and one monomorphic site (at position 740) unique to its own (Table 3; Fig. 2). Quantifying nucleotide differences by AMOVA revealed 32.9% nucleotide variation present between two flax species, 44.5% between pale flax samples from Turkey and those from other countries, and 65% among five groups of cultivated flax. The largest nucleotide difference in cultivated flax was due to the unique haplotype in dehiscent flax samples, and removing dehiscent flax samples generated nonsignificant nucleotide differences among other four groups of cultivated flax.

In summary, the observed nucleotide polymorphism at the locus indicates that cultivated flax has been subjected to a reduced genetic diversity either through a population bottleneck or selection undetected in this study, probably during the domestication process. The genetic diversity is not bilaterally partitioned between pale and cultivated flax, suggesting that the cultivated flax gene pool represents multiple samples of the pale flax gene pool during the domestication process. However, evidence for selection at the *sad2* locus was not strong as revealed with Tajima's *D* or Fu and Li's D* and F* test statistics.

### Phylogenetic network

**Figure 3:**
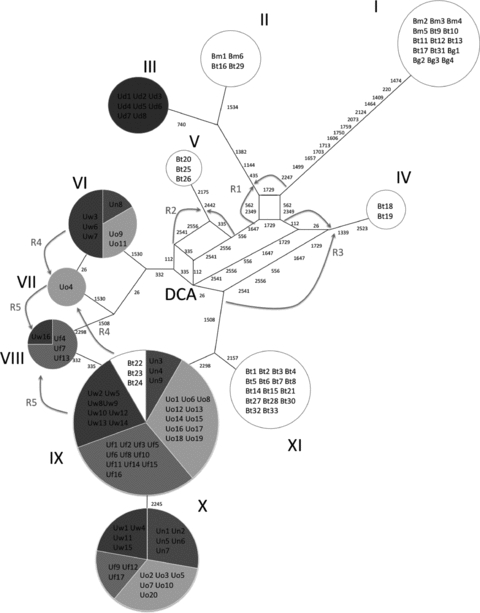
Phylogenetic network of pale flax and cultivated flax at the *sad2* locus. Eleven nodes reflecting 11 haplotypes detected across 113 samples were labeled from I to XI. Labels within a node relate to accessions (see Table 1). The size of node circles relates to sample frequency. The numbers by branches indicate the positions of substitutions detected for that branch. Character conflicts are described as reticulations within the network. The position of the most likely domestication common ancestor (DCA) to all alleles in cultivated flax is indicated by DCA. Five recombination events were detected and numerically labeled.

A network was constructed from 113 samples in this study (Fig. 3). Eleven nodes labeled from I to XI were detected and represented 11 haplotypes across all the samples. The pale flax had five private nodes (I, II, IV, V, XI) and one node (IX) shared with cultivated flax. The largest pale flax node (I), including all four accessions from Greece and some accessions from Turkey and other countries, was distant from cultivated flax, while the others were closely associated with some groups of cultivated flax. The node II of four pale flax samples was closely associated with the node III of eight dehiscent flax samples. The other four nodes (IV, V, X, XI) of 24 pale flax samples collected largely from northern Turkey formed some degree of reticulation with cultivated flax. For cultivated flax, the oil flax occupied four nodes (VI, VII, IX, and X), fiber flax three nodes (VIII, IX, X), and winter flax four nodes (VI, VIII, IX, X). Also, the oil flax appeared to have one private node (VII), while the fiber flax had none. The largest node IX had 36 samples representing the pale flax from Turkey and four groups of cultivated flax (landrace, oil, fiber, and winter). The node X was generated by a nonsynonymous substitution (at the position 2245) and consisted of four groups of cultivated flax (landrace, oil, fiber, and winter). Moreover, the oil and winter flax samples shared one more substitution (at the position 332) with the pale flax samples and seem to be more directly linked to the pale flax from Turkey than the fiber flax. The most likely domestication common ancestor (DCA) detected with the shortest branch length (i.e., with the fewest homoplasies) was consistent with those from the previous analysis with an outgroup (Allaby et al. 2005). It is interesting to note that the dehiscent flax samples from four different countries shared the same distinct genotype, while the locus-specific divergence among other four groups of cultivated flax was not clear-cut.

The network has one large area of reticulation. The recombination analysis (Hudson and Kaplan 1985) performed with DnaSP program revealed at least three recombination events between the sites (26, 332), (335, 1508), and (1729, 2349), respectively. Five recombination events were detected following the methodology of Fu and Allaby (2010) and labeled in Figure 3. The probability of a mutation being homoplasious in the alignment is 1/3*P* (Forster et al. 1996) where *P* = 1/2560 in this study. The network contains 39 substitutions, and the probability of any one mutation being homoplasious in the network is 0.005 [ = (1/3) × (1/2560) × 39], leading to an expectation of 0.19 homoplasies in total. However, a total of seven homoplasies were observed (1.14 × 10^–9^) in the network. For each recombination event, two ancestral nodes are possible, corresponding opposite corners of the associated reticulation. All reticulations were found to be significant at the 5% level, and R3–R5 were significant at the 1% level (Table 4). Our analysis revealed two more recombination events than those detected following Hudson and Kaplan (1985). Therefore, the reticulations in the network largely represent recombination events rather than homoplasies.

**Table 4:** Statistical support for recombination events at the *sad2* locus

Event	*N* (position)^1^	*H*(position)^2^	*N/H*	*P(H/N)*^3^
R1	4 (1382, 1144, 435, 1729)	1 (1729)	0.25	0.02
R2	3 (2541, 2556, 335)	1 (335)	0.33	0.015
R3	4 (112, 26, 1339, 2523)	2 (112, 26)	0.50	0.00015
R4	1 (26)	1 (26)	1.00	0.0051
R5	2 (332, 335)	2 (332, 335)	1.00	0.000025

1 Number of substitutions in branch; substitution position is given in parentheses.

2 Number of homoplasies in branch; substitution position is given in parentheses.

3 Probability of observing *H* or more homoplasies for the given branch length calculated from the binomial distribution.

### Dating flax haplotypes

Pale flax samples were closely associated with groups III and IX, offering some inference of divergence time for these lineages. It seems likely that a group of pale flaxes close to node VI either still exists but has not been sampled or has gone extinct, given the proximity in the network of pale flaxes to the other cultivated groups. The node leading to group III for the dehiscent type of cultivated flax appears to be the deepest in the network for which there are closely related pale flaxes. This suggests that this group is probably the oldest of the cultivated flax groups, which is in agreement with previous EST-SSR data (Fu 2011). Therefore, the node leading to group III makes the most reasonable calibration point using a domestication period of 10,000 yBP (Hillman 1975), which yields a reasonable rate of 3.9 × 10^–8^ subs/site/year (Wolfe et al. 1989). Given this rate of change, the DCA becomes 33,000 years old and the cultivated lineage founded in group IX began roughly 3300 years ago. This rate estimate contrasts with previous estimates (Allaby et al. 2005) in which the DCA node on a much simpler network was used as the basis of a 10,000 years calibration leading to a rate estimate of 1.71 × 10^–7^ subs/site/year, which is about 10-fold higher than would be expected for a plant synonymous substitution rate.

**Figure 4:**
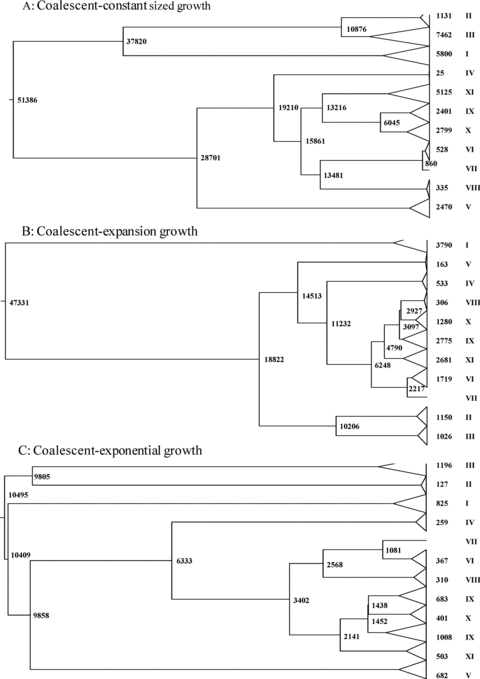
Maximum clade credibility (MCC) phylogenies obtained using BEAST v1.4 (Drummond and Rambaut 2007) with three tree prior models. (A) With tree prior as constant size, (B) with tree prior as expansion growth, and (C) with tree prior as exponential growth. The MCC trees are collapsed for each haplotype (see Fig. 2) that is labeled on the far right column. The ages of the internal nodes, expressed as yBP, are shown on the tree and the ages of the tip nodes on the second right column.

These dates were further investigated with BEAST v1.4, using as a calibration point the II/III group split to a time ranging from 11,000to 9500 yBP. Three MCC trees obtained using BEAST v1.4 are shown in Figure 4. Interestingly, the three models correctly identified each member of 11 haplotypes with one exception and generated similar topologies that are compatible with the network shown in Figure 3. The exception occurred under the exponential expansion model where groups IX and X become an unresolved polytomy, which is a minor deviation in topology due to the effects of recent expansion. The topology of the constant sized population matched the topology of the network most closely, while the expansion growth model placed group VIII as a sister taxon to group X, and the exponential growth model failed to resolve groups IX and X. The ages of the nodes are shown on the trees expressed as yBP. The age estimates obtained overlap with the estimates from the network in the case of the constant population size and expansion models. The exponential model, however, poorly fitted the data with the formation of the dehiscent and associated pale flax clade being close to the root of the entire tree despite only two substitutions along these branches. Under the constant population size model, the origin of the IX lineage is around 6045 yBP, and the DCA node is dated to around 15,861 yBP with a wide margin of error extending to around 47,000 yBP. The expansion model, which is perhaps more likely to reflect the true underlying population process for cultivated flax at least, yielded younger dates, with the IX lineage being around 3097 yBP, which is close to the network estimate. In this case, the DCA node is very young at around 6247 yBP, also with a wide error extending to 26,000 yBP. In reality, it is likely that the true underlying population process that gave rise to this phylogeny would have been a combination of long-term constant population size for the pale flax populations, followed by an expanding cultivated population. The existence of group XI suggests that the DCA node would have been represented more likely by pale flax rather than cultivated flax. Consequently, the DCA node probably relates to a time before an expansion process associated with cultivation would have taken place, leading to the optimum date under the expansion model of 6247 years probably being inappropriate.

### Flax domestication history at the sad2 locus

The level of unsaturated fatty acids in flax seeds increased during domestication involving an apparent increased productivity of the *sad2* locus, indicating that the *sad2* locus may be considered a candidate domestication locus. However, the mechanism of increased productivity has not yet been discovered. It is highly possible that the *sad2* locus may not be the only candidate locus, as other cis- and trans-acting loci may have been involved with the fatty acid metabolism. Thus, the true contribution of the *sad2* sequence variation to the difference observed in fatty acid composition between the cultivated and pale flax samples remains unknown. Either way, the phylogenetic reconstruction of the *sad2* locus reflects a specific history associated with increased unsaturated oil production in cultivated flax.

The network analysis involving a large set of pale flax and four groups of cultivated flax revealed a complex domestication history of flax that has not been previously observed. The pale flax displayed two different groupings in agreement with other studies (Uysal et al. 2011). One group represents two lineages (I and II) including pale flax samples collected from different countries including western Turkey and Greece and has an indel shared with the dehiscent type of cultivated flax (III). Comparison with the *sad1* sequence, which does not have the deleted character state, indicates that this indel is a deletion in the branches leading to groups I to III rather than an insertion in groups IV and above. The second group (XI) represents only the pale flax samples from northern Turkey along the Black Sea coast and has a genetic background shared more closely with the indehiscent groups of cultivated flax (VI, VII, VIII, IX, and X). These genetic associations expand on the previous observation of the basal position of the dehiscent flax group (Fu 2011). These data indicate that the dehiscent cultivated flax lineage should be regarded as an independent domestication. The molecular dating used in this study confirms the early nature of this domestication, before the subsequent domestication process that led to the indehiscent cultivated flax groups. Interestingly, the oil profile data (Table 2) indicate that the increase in unsaturated fats had occurred in the dehiscent cultivated flax lineage, but loss of seed dispersal through capsular indehiscence had not. This suggests that selection for oil composition came before loss of seed dispersal, and that the dehiscent cultivated flax lineage represents an alternative or incomplete domestication trajectory as compared to the other cultivated flax groups. This order of trait fixation is similar to the case of cereals in which loss of seed dispersal was a trait that was fixed late in the domestication process (Tanno and Willcox 2006; Fuller 2007).

The indehiscent cultivated flax groups appear to represent a domestication process that may have involved more than one domestication. The close proximity of group IX to the pale flax group XI suggests that this is a separate domestication to that associated with group VI. An alternative explanation could be that the indehiscent cultivated flax was domesticated from a genetically diverse population (Charlesworth 2010) that has maintained two distinct lineages. However, the distinct geographical clustering of the pale flax suggests that the pale flax populations tend not to be so diverse, making this is a less likely explanation based on current evidence.

Oil flax varieties occur in both the indehiscent cultivated flax lineages, but fiber varieties appear to be restricted to the IX–X lineage. Note that group VIII, which includes fiber varieties, was formed through a recombination event between groups IX and VII, so these fiber accessions should be considered as part of the IX–X lineage. This phylogenetic restriction suggests that flax was used for oil before fiber, which agreed to previous studies (Allaby et al. 2005; Fu and Allaby 2010). If the *sad2* locus was directly responsible for the increase in unsaturated oil composition, then the phylogenetic pattern in Figure 3 suggests that parallel changes happened in the dehiscent cultivated flax and indehiscent cultivated flax groups. The data support multiple independent pathways of domestication of flax for oil composition. Therefore, it is likely that fiber varieties evolved from a lineage of flax domesticated for oil. In support of this scenario, the oil profile data indicate that fiber flax also showed increased unsaturated fatty acid content despite its usage, suggesting that this is a vestigial feature of fiber flax. The dating analysis further supports this scenario, with an origin of the fiber lineages occurring around 3000 years ago.

### Domestication-associated locus-specific analysis

The analysis of the *sad2* locus revealed a complex history of cultivated flax that is informative about the origins of oil, fiber, and dehiscent varieties despite the apparent restriction that the locus is specifically associated with the oil production. The results expand on, rather than conflict with, earlier studies (Allaby et al. 2005; Fu 2011). It may be relevant that the trait of oil composition is clearly primary, and so underlies all the flax varieties which all have the trait despite not necessarily being exploited for it. However, it is less clear what is revealed about winter tolerance. The winter tolerant varieties were not topologically restricted in the network as in the case of fiber varieties. It therefore seems likely that winter tolerance preceded fiber production in these lineages, but we have no resolution between oil production and winter tolerance in the indehiscent cultivated flax samples. The oil profile data demonstrate that oil production has been enhanced in all varieties making it much more likely that this domestication-associated trait occurred before winter tolerance. It was not until flax spread from the Near East into the Danube valley some time after the initial domestication that winter tolerance was required (Helbaek 1959; Diederichsen and Hammer 1995). In this case, it is likely that increased phylogenetic resolution could be obtained through the study of a locus specifically associated with winter tolerance.

We suggest that there are some general principles for domestication-locus specific studies, which should be considered in future studies. First, the domestication processes influenced flax traits, and loci governing different traits may have different patterns of genetic diversity, depending on different selection processes and the underlying genetics of the target traits (Fu 2011). Thus, it is important to analyze as many domestication loci as possible to infer the processes in which domestication traits were acquired by cultivated plants. This is, particularly true for the candidate domestication loci without direct function evidence. The *sad2* locus is a candidate domestication locus, but not necessarily the most informative one. Inferences based on other related candidate loci such as the *fad* loci (Banik et al. 2011) may help to expand the historical view presented here. Second, a direct inference of causative domestication loci should always be encouraged for high resolution. However, most loci governing domestication traits are not cloned and sequenced in crops such as flax and so may not be accessible to such inference (Fu and Peterson 2010), which thus limits the power of the locus-specific analysis (Konishi et al. 2008; Blackman et al. 2011). Third, the pattern of genetic diversity in an influenced locus depends in part on the degree of human-mediated selection that has acted on various target traits, and any single locus may capture a variable domestication signal. In this case, the *sad2* locus may carry more information on oil selection than the other domestication traits of fiber production, winter habit, and dehiscence, so a bias toward oil selection could exist.

## Conclusion

The domestication-associated locus-specific analysis in this study has revealed a complex picture of flax domestication involving multiple paths of domestication, initially for oil. An independent alternative or incomplete domestication trajectory occurred in the dehiscent flax group in which the loss of seed dispersal did not occur. It may be the case that the human-mediated selection pressures were different for these plants than for the indehiscent cultivated flax. Furthermore, a recent origin of fiber varieties is apparent, probably in the order of 3000 yBP. Consequently, it is apparent that despite being a locus that is associated with oil rather than fiber or dehiscence, *sad2* has been informative to a degree for more than just oil varieties. However, it is clear that there is a limit to the resolution achievable in that little could be resolved about winter tolerance other than it occurred prior to fiber varieties, and the locus-specific approach would be enhanced by considering more loci relevant to the other traits also, and within the wider context of genome-wide information.
